# Special Issue: Celebrating the Career of Donald F. Hunt

**DOI:** 10.1016/j.mcpro.2025.101468

**Published:** 2025-12-30

**Authors:** Jeffrey Shabanowitz, Jennifer G. Abelin

**Affiliations:** 1 Department of Chemistry, University of Virginia, Charlottesville, Virginia, USA; 2 Proteomics Platform, Broad Institute of MIT and Harvard, Cambridge, Massachusetts, USA

## Preface

In this special issue of *Molecular and Cellular Proteomics*, we wish to recognize and celebrate the career achievements of Professor Donald Frederick Hunt upon his retirement after 60 years in science. The articles included present a chronological journey through his scientific career with contributions from former graduate students, postdoctoral fellows, colleagues, and long-time friends. They also represent a tribute to the impact Don has had on the field of mass spectrometry: from applying chemistry and developing instrumentation, to solving analytical and biological problems, to training future scientists. As an editor of this issue, I offer first my personal, heartfelt remembrances of my life spent with my mentor, my colleague, and my friend. Thank you for everything, Don.

### Prologue

It was a dark and stormy night…well, actually, it was lunchtime in Frank Field’s lab at Rockefeller University in NYC, one day in late September 1975. As I settle down to eat lunch at my desk and peruse the latest issue of *Chemical & Engineering News*, I notice at the back a two-line ad for an academic position, something to the effect of, “Research Assistant to work on negative ion chemical ionization, contact Donald F. Hunt, University of Virgina.” Well, I have now been with Frank Field for 3.5 years and while I am and always will be grateful for the opportunity to learn mass spectrometry from Frank, I am feeling impatient to go back to school and pursue my PhD. After all, by this time I am training post-docs new to Field’s lab to operate and maintain the DuPont 21−492 with a custom-in-house chemical ionization (CI) source and realize I am ready. After reading a bit more of what the Hunt Lab was up to, I thought, “What better way to pursue my degree than to go from one state-of-the-art mass spec lab pioneering chemical ionization to another applying chemical ionization to negative ions?” So, I speak to Frank about it, and he gives his blessing, he contacts Don on my behalf, and the next thing you know I’m on a Piedmont flight from LaGuardia to Charlottesville.

Okay: now fast-forward 50 years: as I’m writing this preface for this *MCP* Special Issue to honor Don, I realize that unbeknownst to me at the time, the events I’m relating were to be the beginning of the next half century of my life.

I get off the plane in Charlottsville and find that there is one runway, one gate, and a 3-foot-high chain-link fence surrounding the airport (Oh, how the times have changed!). Don is there to pick me up with his 2-year-old daughter Caroline in his old green Volvo station wagon with a standard shift. (Those of you who remember Don’s Massachusetts driving habits, will also remember that he only drove stick shifts up until perhaps 20 years ago when they were no longer being offered.) So off we go to town, not down Route 29 but rather down the backroad Route 743 or Earlysville Road. Those familiar with the area know the Earlysville Road to be a narrow, winding, two-lane road, and those familiar with Don’s driving habits know that Don drives at one speed: whatever the speed limit is. In this case, it was 55 mph. While he’s driving and shifting, there beside me is tiny Caroline who is petrified. She has never seen anyone as big as me, and she is trying to get as far away from me as she can (note that this story obviously predates mandatory seat belts and children’s car seats). Anyway, she is clamoring to get away from me and climbs into Don’s lap. Without missing a beat or slowing down, he carefully picks her up with both hands, deposits her in the console space, and calmly says, “Caroline, please, I’m driving. He won’t hurt you.” …and yes, he was driving at 55 mph on a winding country road, steering with his knees while he tended to his terrified daughter.

And that’s where this story begins. It was touch and go as to whether or not I would take the position. Both Don and the UVA Chemistry Department wanted me. The position being offered was a joint one, running the two departmental mass spectrometers: a Hitachi RMU-6e (single focusing) and an AEI MS-902 high resolution mass spec with a chemical ionization (CI) source, as well as Don’s Finnigan 3200 single quadrupole GC/MS modified for PPINICI (Pulsed Positive Ion Negative Ion Chemical Ionization; https://doi.org/10.1016/j.mcpro.2025.101033). I was hesitant because I would be replacing a person who was fired because neither of the department mass spectrometers was working. Did I want to walk into a potential minefield? I was confident enough in my abilities with instruments to think I could fix the broken ones, but then there were the hundreds of backlogged samples, and the disgruntled faculty wanted their samples run yesterday. It was Don who, in his usual quiet, mild-mannered fashion, sweet-talked me into accepting the challenge.

### The Early Years

After the first year, with both departmental instruments working again and the backlogs cleared, I was able to contribute to Don’s research endeavors. I worked closely with George Stafford in his last year at UVA before he went to Finnigan (Fall 1976). Don then ‘flipped the script’ with the department and supported me at 90%, while the department kicked in 10% so I could oversee a graduate student tasked to run the service samples. Now I was really part of Don’s team.

In the spring of 1978, Don heard a talk by graduate student Rick Yost at the annual mass spectrometry conference and saw the Yost and Chris Enke JACS paper of that year. This work demonstrated MS/MS on small hydrocarbons with high efficiency using a tandem quadrupole mass spectrometer (TSQ) vs the MIKES (mass-analyzed ion kinetic energy spectrometry) experiments on the magnetic sector instruments. Don’s comment to me: “Why are they doing *that*? This can be “super” (Don’s favorite word) for getting structural information on peptides and small molecules! Jeff, can we build one of these?” “Umm…yes, Don,” I replied somewhat hesitantly. “But I would need components from the other instruments in the lab and if I take those no one else will be able to do any research.” Don’s response? “DO IT!”

As of summer 1978, the lab had one single quad with PPINICI and two single quad Finnigan GC/MS instruments. By combining parts from these instruments with additional parts made in the UVA Chemistry Department machine shop and some components from Finnigan, I had everything I needed. Thus, on a Monday morning in July, all the Hunt lab instruments were shut off (to the horror of the other unsuspecting students and post-docs in the lab!), the disparate pieces were quickly pulled together and assembled, and by Friday we had obtained the first MS/MS spectrum of a small peptide from our newly constructed TSQ. Don quickly sent a copy of the spectrum to Chris Enke with the simple acknowledgement, “It works!”

The Chemistry department finally accepted me as a part-time graduate student in the Fall of 1978. I had proved that I could pass some of their mandatory classes but also by then I had garnered Don’s full support. After demonstrating the potential of TSQ technology in a variety of areas, one of the first practical applications was to analyze and identify compounds on the EPA’s priority pollutants (PPs) list. The EPA-authorized method for analysis of environmental samples for these compounds was GC/MS (typically on Finnigan single quads). Don contacted a representative of the USEPA and challenged them to bring us test samples containing priority pollutants that we (read I) would analyze in less than a day. The speed with which we promised (boasted) to obtain results stood in sharp contrast to the time such an analysis would have required by conventional GC/MS methods. The samples were brought, and I somehow managed to analyze them in time to present results by 5 PM the same day. We identified most of the PPs correctly in a fraction of the time and at what would have been a fraction of the cost, per “Don’s math”. However, our efforts were to no avail as the EPA was already invested in the standard protocols using GC/MS for this analysis and being a government entity, this was not something easily changed.

### Don Makes it Snow

Don spent his sabbatical of the academic year 1981 to 1982 in Howard Morris’ lab at Imperial College London to learn more about protein sequencing by MS. When he left, our lab consisted of 16 students and post-docs all working on different projects Don had suggested. However, on his return, none of these projects was of great interest to him. He was suddenly hell-bent on establishing a peptide/protein sequencing lab. Over the next couple of years, the existing graduate students and post-docs finished their projects and moved on. I graduated in 1983 and, although I had several opportunities to go elsewhere, Don convinced me to stay. A new era was dawning in the lab and by the mid-1980’s three graduate students, John Yates, Pat Griffin, and Charles Hauer, and I were all committed to Don’s transformed vision for the lab.

Thanks to the work of Mickey Barber at the University of Manchester, we were armed with fast atom bombardment (FAB) to ionize the biological entities, but we needed something more: an instrument with high resolution, higher mass range, and better sensitivity. Thus, the Quadrupole-Fourier Transform mass spectrometer (QFTMS) idea was hatched. Unbeknownst to us, Robert McIver (UC Irvine) was simultaneously pitching the very same idea to Finnigan. In 1984, with help from Finnigan, we procured the magnet and built a prototype instrument (https://doi.org/10.1016/j.mcpro.2024.100866).

On a Monday, a couple of years after the instrument was in use, I awoke with a 24-h flu that left me immobilized (I had never felt so bad in my life; I truly thought I was going to die). Being Monday morning, the QFTMS with its 7 T magnet needed to be fed its liquid nitrogen (LN2) diet to prevent its quenching. A dose of LN2 was required every 5 days without fail. I called the lab to say I wouldn’t be in, so someone else would need to feed the magnet. Don said, “Don’t worry. I can do it.” I was so sick I didn’t argue.

There was enough LN2 in the dewar that all Don had to do was roll it close to the magnet and hook up the ∼10′ transfer line. As Don rolled the dewar towards the magnet, he turned to get the transfer tube. The dewar was still moving however, and as it came into the magnet’s field, its speed accelerated, which sent it into the corner of the magnet. Don claims he tried to stop it, but the dewar was within the full grasp of the magnetic field, and Don was lucky not to have been crushed between them.

What came next only Don can adequately describe. The magnet quenched, dumping the 60 amps that charged the 7T superconducting coil instantly, vaporizing all the LN2 and liquid helium (used to maintain the field) and making it snow in the lab. He then had to call me and confess what had happened; I was so sick that my reaction was probably not what he expected. All I said was, “Okay, go down to Paul Schatz’ lab and see if his student who takes care of Paul’s magnets is there. If she is, ask her to get more LN2 and fill the magnet so that at least it will stay cold and not condense moisture inside. I will assess the damages when I’m able to come in.”

The next day when I got in, I could see the abrasion on the corner of the magnet. My fear was that the superconducting coils had shorted when the magnet quenched. Miraculously, with true Don luck, they were okay. By Wednesday I had the field back up and by the end of the week I was able to check for ions. To my very great surprise, the QFTMS actually seemed to behave better and have more sensitivity than before the accident. To this day Don will claim that he was “tuning the instrument!”

### Letting Application Drive Innovation

As Don won award after award in the late 1980s and early 1990s, it was inevitable that he would be offered opportunities to leave Charlottesville. In 1992, Emil Unanue invited him to come to Washington University in St Louis. His offer included half a floor in a new building, a ridiculous (for the time) amount of startup money, and large salary bumps. I knew about the terms of the offer and was impressed. What I didn’t know was that Don took the offer to the University of Virginia and dangled before the administration the possibility of our leaving. Luckily, UVA countered: they gave Don a joint appointment in the Medical School, made him University Professor, granted me non-tenured faculty status, and we all got nice salary bumps; hence, here we stayed. Years afterwards when I learned of Don’s ploy, I asked him, “What were you going to do if UVA had said no? Because I sure as heck didn’t want to move to St Louis.” He said he knew that and in fact neither did his wife Linda, so he was just hoping UVA would counter. They did, and the rest, as they say, is history.

With electrospray ionization (ESI) now available, it was time to get serious about interfacing liquid chromatographs (LC’s) to the mass spectrometers. The LC’s and columns at the time were large, off-line, high flow, high pressure, high theoretical plate systems built for maximum separation. In contrast, Jim Jorgenson at UNC was developing micro separation systems for capillary electrophoresis (CE) and LC. Don persuaded Jim to send one of his graduate students, Arthur Moseley, to our lab to show us their technology. Arthur shows up with his meter-long, self-packed HPLC columns built for exquisite separation: a huge number of theoretical plates and flow rates at sub-μL’s per minute into the ESI source. He and I set up the experiment and waited for the peptides to elute…for the first hour, then the second hour… Arthur is unphased by the wait: he knows his system is working and this is normal. Now it is lunch time so we go to the Corner, get something to eat, and come back to the lab. The system is still running but nothing has eluted.

I finally say to Arthur, “Something has to be wrong, and besides it’s going to take 3 h to do one experiment.” I know Don is not going to be happy. We shut off the HPLC and, true to form, the first peptide starts to elute and gives a beautiful signal. It is also at this moment that Don shows up, asks how the experiment is doing, and we fill him in. He takes a long look at the set up. Arthur hands him one of his painstakingly self-packed, 1-m-long columns to examine. Just like that, Don snaps it off at about 6 inches and says, “Here ya go! That’s all we need. We don’t need a million theoretical plates for separation; we have a mass spectrometer and can separate by mass.” The shock on Arthur’s face was something else, but so were the experiments that we ran for the next few days on those 6” columns! And with that, our LC/ESI/MS studies of peptides were off and running.

Once the chromatography was sorted, Don’s interests turned to immunology. In collaboration with Vic Engelhard (UVA Microbiology and Immunology Dept) (https://doi.org/10.1016/j.mcpro.2024.100823) we set about transforming the way Major Histocompatibility Complex (MHC) peptides were sequenced. It took Vic almost a year to get an MS-compatible sample of MHC class I peptides. Once we had that, we were able to obtain high-resolution MS1 spectra on the QFTMS. Another example of “Don math”: after he had me print out all the MS1 scans during the 20 min that peptides eluted from the LC, he took that stack of printouts and manually counted the signals to estimate how many peptides were being presented on the cell surface. The number he came up with from his counting − ∼60,000, or ∼10,000 per allele – still stands today.

In the early 2000’s, Don’s interest in PTMs expanded to the histone and glycobiology fields (https://doi.org/10.1016/j.mcpro.2024.100873 and https://doi.org/10.1016/j.mcpro.2024.100859). The problem was site-localization. Don saw what ECD (Zubarev/McLafferty) was capable of and came to me with the desire to install it on the LTQ-FTMS. I told him, “No, you don’t want that,” he asked why, and I said, "It takes too long!”- at the time the thermal electron source only allowed for one ECD spectrum every 1 to 2 min-not a chromatographic timescale. He then turned to John Syka and asked about trapping electrons in the ion trap, which of course you can’t do but then Jarrod Marto piped in, “What about ion-ion reactions?” And with that, we were off and running once again. Josh Coon had just joined the group as a post-doc with an NIH fellowship to explore atmospheric MALDI on an ion trap. When Don first approached him to work on ion-ion reactions with John, Josh declined citing the goals of his fellowship. In true Don fashion, he said to him, “Oh don’t worry about that. They don’t care what you do as long as it’s good science and this will make your career!!!” We had gone from using ion molecule reactions for chemical ionization to solve small molecule problems to using ion-ion reactions on highly charged peptides and proteins. The next step was inevitable, using ETD and PTR to completely characterize and sequence intact proteins (https://doi.org/10.1016/j.mcpro.2025.100943).

### Epilogue

As the years passed and the lab’s research areas evolved, we were rarely the first to do something, but Don had a knack for immediately grasping the potential of others’ innovations, often seizing on their applicability before their developers had time to brainstorm. He and I didn’t always agree, but with his insatiable curiosity and unending list of ideas and my strong desire to please him, we managed to accomplish a lot. As the papers in this special issue will attest, his vision expressed as short-term and long-term goals has kept us busy for fifty wonderfully productive years.

We would like to thank everyone for their hard work and efforts to make this tribute possible. Steve Carr and MCP, who first approached me with this idea a year ago and Jeanne Gladfelter (MCP) for her assistance along the way. Special thanks to Jane Gale for her astute advice and help along the way. This was indeed a labor of love for everyone involved. Thank you all!

Jenn Abelin and Jeff Shabanowitz

The Academic Family Chemistry for Donald F. Hunt and list of publications can be found at https://academictree.org/chemistry/peopleinfo.php?pid=63541

A timeline of Donald F. Hunt and the Hunt laboratory major milesones can be found in Jessica R. Chapman’s chapter in this issue-https://www.sciencedirect.com/science/article/pii/S1535947624001567?via%3Dihub#fig3

Tributes and memories to Don Hunt can be found at – https://docs.google.com/document/d/1cfpLJ4hY6NNvbn2QL_0461mzqG7Z4Glxq2YBCuVHAv4/edit?tab=t.0

Please send requests to add to these to myself at jeffreyshab@gmail.com or Jenn Abelin at jenn.abelin@gmail.com

